# Supplementary material for: Integrated analysis of the aqueous humor microbiome and lens capsule transcriptome in high myopia cataract: a pilot study
Source: Front Med (Lausanne). 2026 Jun 16;13:1845205. doi: 10.3389/fmed.2026.1845205 (PMC13314463; doi:10.3389/fmed.2026.1845205)
Supplement: Supplementary file 3 [file Data_Sheet_1.zip › 2.Alpha_Diversity/alpha_rarefaction_plot/C372089/simpson.pdf]

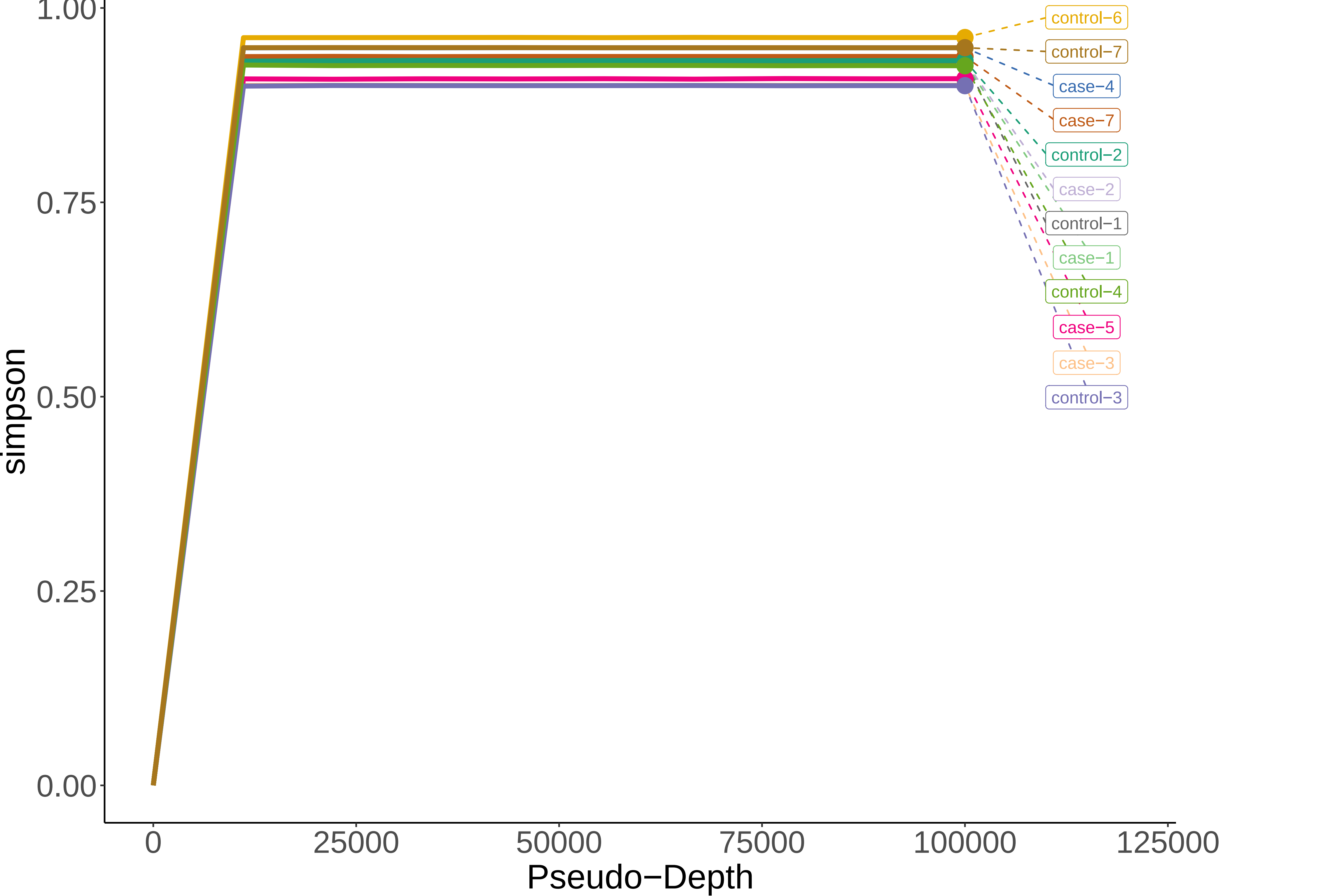
1.00
0.75
0.50
0.25
0.00
simpson
0
25000
50000
75000
100000
125000
Pseudo−Depth
control−6
control−7
case−4
case−7
control−2
case−2
control−1
case−1
control−4
case−5
case−3
control−3
